# Anisotropic Thermal Expansion in an Anionic Framework Showing Guest-Dependent Phases

**DOI:** 10.3389/fchem.2020.00506

**Published:** 2020-06-18

**Authors:** Zhu Zhuo, You-Gui Huang, Krista S. Walton, Osamu Sato

**Affiliations:** ^1^CAS Key Laboratory of Design and Assembly of Functional Nanostructures, and Fujian Provincial Key Laboratory of Nanomaterials, Fujian Institute of Research on the Structure of Matter, Chinese Academy of Sciences, Fuzhou, China; ^2^Xiamen Institute of Rare Earth Materials, Haixi Institutes, Chinese Academy of Sciences, Xiamen, China; ^3^School of Chemical & Biomolecular Engineering, Georgia Institute of Technology, Atlanta, GA, United States; ^4^Institute for Materials Chemistry and Engineering, Kyushu University, Fukuoka, Japan

**Keywords:** thermoresponsive, anisotropic, anionic framework, host–guest interaction, phase

## Abstract

Crystalline materials generally show small positive thermal expansion along all three crystallographic axes because of increasing anharmonic vibrational amplitudes between bonded atoms or ions pairs on heating. In very rare cases, structural peculiarities may give rise to negative, anomalously large or zero thermal expansion behaviors, which remain poorly understood. Host–guest composites may exhibit such anomalous behavior if guest motions controllable. Here we report an anionic framework of helical nanotubes comprising three parallel helical chains. The anisotropic interaction between the guest and the framework, results in anisotropic thermal expansion in this framework. A series of detailed structural determination at 50 K intervals enable process visualization at the molecular level and the observed guest-dependent phases of the framework strongly support our proposed mechanism.

## Introduction

Actuators based on materials that reversibly change shape and/or size in response to external stimuli are highly desirable (Otsuka and Wayman, 1998; Yu et al., 2003). Photo-responsive materials, which enable remote operation without direct contact with the actuators, have attracted particular interest, some exceptional cases have also been shown (Kobatake et al., 2007; Kumpfer and Rowan, 2011). Most of these materials exploit the photo-isomerization of constituent molecules, which induces molecular motion and thereby deforms the bulk material (Kobatake et al., 2007; Nabetani et al., 2011; Yamaguchi et al., 2012). Thermal expansion also exemplifies the impact of external stimuli on material properties. Indeed, solid-state materials typically expand along all three crystallographic axes with increasing temperature (positive thermal expansion (PTE), 0 < α < 20 × 10^−6^ K^−1^, where α is the axial thermal expansion coefficient), however a few materials show little response (zero thermal expansion (ZTE), α ≈ 0 K^−1^) or shrink (negative thermal expansion (NTE), α < 0 K^−1^) along a specific crystallographic direction upon heating (Salvador et al., 2003; Goodwin et al., 2008; Long et al., 2009; Greve et al., 2010; Phillips et al., 2010; Azuma et al., 2011; Fortes et al., 2011; Yamada et al., 2011; Huang et al., 2013). Materials exhibiting ZTE or NTE are technologically useful in areas such as heat-engine components, structural engineering applications, and thermomechanical actuators or sensors, etc (Peter et al., 2011). Characteristic NTE-type materials include a small number of inorganic oxides and zeolites (Evans, 1999; Lightfoot et al., 2001), a family of cyanide coordination frameworks (Goodwin and Kepert, 2005; Goodwin et al., 2005, 2008; Korčok et al., 2009) and some organic compounds (White and Choy, 1984; Birkedal et al., 2002; Das et al., 2010). Goodwin et al. have shown that Ag_3_[Co(CN)_6_] exhibits “colossal” positive and negative thermal expansion by flexing like lattice fence (Goodwin et al., 2008). Barbour et al. have reported that (*S,S*)-octa-3,5-diyn-2,7-diol displays exceptionally large positive and negative anisotropic thermal expansions because the molecules pack down on heating (Das et al., 2010). de Pedro et al. have observed colossal and highly anisotropic thermal expansion on several imidazolium salts (de Pedro et al., 2015). Very recently, some metal-organic frameworks (MOFs) have shown such anomalous thermal expansion behavior, which usually stems from a hinged movement around the metal center (Wu et al., 2008; Zhou et al., 2008; Miller et al., 2009; Yang et al., 2009; de Vries et al., 2011; Grobler et al., 2013; Shang et al., 2014; Pang et al., 2016). Elucidating the mechanisms governing this behavior becomes a fascinating subject because it offers information on new design principles for thermoresponsive materials. Host–guest composites may provide good examples of NTE-type materials and the mechanisms can easily be rationalized at the molecular level if guest motion can be visualized and controlled. The state of guest molecules confined in a host is expected to be readily modified under extra stimuli, which may drive the host to respond. For instance, Chen et al. have directly visualized the crystal deformation of a MOF triggered by guest rotation (Zhou et al., 2013). The challenge is rationally translating guest motion into crystal lattice deformation because the thermodynamic energy changes of these flexibilities usually differ significantly in magnitude. To address this issue, a flexible porous anionic framework with counter cations strongly confined by hydrogen bonds was designed. Sites interacting with the guest may perceive guest motions and properly transfer these local molecule motions into bulk mechanical response because of the flexibility of the confinement.

## Experimental Section

### Materials

All reagents, except H_4_BPTC, were purchased from commercial sources and used without purification.

### Synthesis

H_4_BPTC: H_4_BPTC was synthesized by a modified literature procedure (Lin et al., 2009). 3,3′5,5′-tetramethylbiphenyl (1.0 g, 0.0047 mol) was oxidized using KMnO_4_ (6.5 g, 0.112 mol) in tert-butanol/water (v/v = 1:1; 50 mL) containing NaOH (0.4 g, 0.01 mol). Yield: 1.12 g 72.2%. Anal. Calcd (Found) for C_16_O_8_H_10_: C, 58.19 (58.10); H, 3.05 (3.09) %.

[Me_2_NH_2_]·[Mg_2_(BPTC)(NO_3_)(H_2_O)] (**1**) A mixture of Mg(NO_3_)_2_·6H_2_O (0.052 g, 0.2 mmol), H_4_BPTC (0.033 g, 0.1 mmol), 4,4′-azopyridine (0.018 g, 0.1 mmol) and HCl (1 mL 1mol/L) in DMF (5 mL) was heated at 120°C in a sealed 20 mL glass via for 1 day, and cooled to room temperature. Colorless needle crystals of compound **1** were filtrated, washed using methanol and air-dried (0.041 g, Yield: 80% based on Mg). Anal. Calcd (Found) for MgNC_9_O_6_H_8_: C, 43.12 (43.03); H, 3.19 (3.11); N 5.59 (5.63)%.

[EtNH_3_]·[Mg_2_(BPTC)(NO_3_)(H_2_O)] (**2**) A mixture of Mg(NO_3_)_2_·6H_2_O (0.052 g, 0.2 mmol), and H_4_BPTC (0.033 g, 0.1 mmol) in NEF (5 mL) was heated at 120°C in a sealed 20 mL glass via for one day, and cooled to room temperature. Colorless needle crystals of compound **2** were filtrated, washed using methanol, and air-dried (0.035 g, Yield: 70% based on Mg). Anal. Calcd (Found) for MgNC_9_O_6_H_8_: C, 43.12 (43.06); H, 3.19 (3.09); N 5.59 (5.51)%.

### Measurements

Elemental analyses were performed using a Vario EL elemental analyzer. IR (KBr pellet) spectra were recorded in the range of 400–4,000 cm^−1^ on a JASCO FT/IR-600 Plus spectrometer. Thermogravimetric analyses (TGA) were performed using a TG/DTA6300 system at a rate of 5°C/min under N_2_ atmosphere. Powder X-ray diffraction (PXRD) patterns were acquired using a Rigaku 2100 diffractometer with Cu *K*_α_ radiation in flat plate geometry. The temperature increased at a rate of 10°C/min and was held constant for 5 min at each targeted temperature before measurement.

### Single-Crystal X-Ray Diffraction

Diffraction data were collected on a Rigaku-CCD diffractometer with Mo *K*_α_ radiation. The temperature was changed at a rate of 10°C/min and was maintained at each targeted temperature for 5 min before measurement. Structures were solved by direct method and refined by full-matrix least-squares analysis on *F*^2^ using the SHELX program. Hydrogen atoms were generated geometrically and refined in a riding model. For compound **1**, anisotropic thermal parameters were applied to all non-hydrogen atoms. Guest cations in compound **2** were isotropically refined. NO_3_^−^ and guest cations in compound **1** as well as guest cations in compound **2** were restrained by several DFIX instructions. The occupancies of μ-NO_3_^−^, μ-H_2_O and guests in all structures were refined to be consistent with the results of elemental analyses.

## Results and Discussion

**Figure 1:**
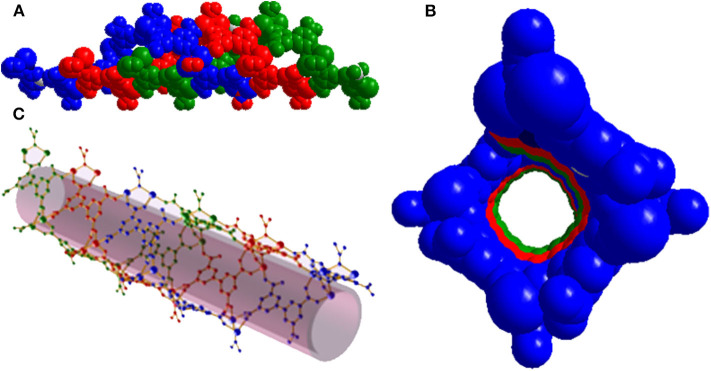
**(A)** Side view of the helical nanotube of triple helices in compound **1**. **(B)** Top view of the helical nanotube. **(C)** Perspective view of the helical nanotube. Helices are differentiated by their colors (blue, red, and green).

The solvothermal reaction of Mg(NO_3_)_2_·6H_2_O, biphenyl-3,3′5,5′-tetracarboxylic acid (H_4_BPTC) and 4,4′-azopyridine (AZPY) in dimethylformamide (DMF) afforded [Me_2_NH_2_]·[Mg_2_(BPTC)(NO_3_)(H_2_O)] (**1**) as colorless needle-shaped crystals. Single-crystal X-ray crystallography revealed that compound **1** consists of a three dimensional (3D) chiral anionic framework assembled from nanotubes comprising parallel triple helices. Charge neutrality is achieved by protonated dimethylamine cations [Me_2_NH_2_]^+^. NO_3_^−^ anions and H_2_O molecules present a statistical distribution in the anionic framework (Figure S1). Therefore, the formula of compound **1** was determined by combining single-crystal X-ray crystallography, thermogravimetry (TG) and elemental analysis, results. Compound **1** crystallizes in the chiral orthorhombic space group *I*2_1_2_1_2_1_ and the asymmetric unit contains a Mg^2+^ ion, half a BPTC^4−^ ligand, half a μ-NO_3_^−^ anion and μ-H_2_O in statistical distribution and half a [Me_2_NH_2_]^+^ cation. Each Mg^2+^ ion is coordinated with six O atoms in an octahedral geometry, four of which from four different BPTC^4−^ ligands and the other two from two different μ-NO_3_^−^ or μ-H_2_O (Figure S2). All four carboxylate groups of BPTC^4−^ ligand adopt a μ_2_-η_1_-η_1_ bridging mode to ligate to two Mg^2+^ ions (Figure S3). Two Mg^2+^ ions are bridged by μ-NO_3_^−^ or μ-H_2_O to form {Mg}_2_ dinuclear units which are further linked together by BPTC^4−^ ligands giving rise to a helical chain running along the crystallographic *a* axis (Figure S4). Three homochiral helices associate in parallel to generate the wall of a helical nanotube with an opening of about 11 × 11 Å^2^ (Figure 1). As mentioned above, the Mg^2+^ ions favor a hexacoordinated environment, while five of these sites are dedicated to the nanotube, the remaining one provides an additional binding site for nanotube assembly into a 3D array. Specifically, each nanotube serves as a tertiary building unit and is further linked to its four adjacent neighbors to generate a 3D chiral anionic framework. The [Me_2_NH_2_]^+^ cations stacking in helical template are confined in the nanotube strongly by hydrogen bond formation with a BPTC^4−^ ligand O atom in the framework (Figure S5). As a result, the channel is predominantly obstructed leaving an effective void volume of only ~12.3% as [Me_2_NH_2_]^+^ cations could not be removed by simple activation.

**Figure 2:**
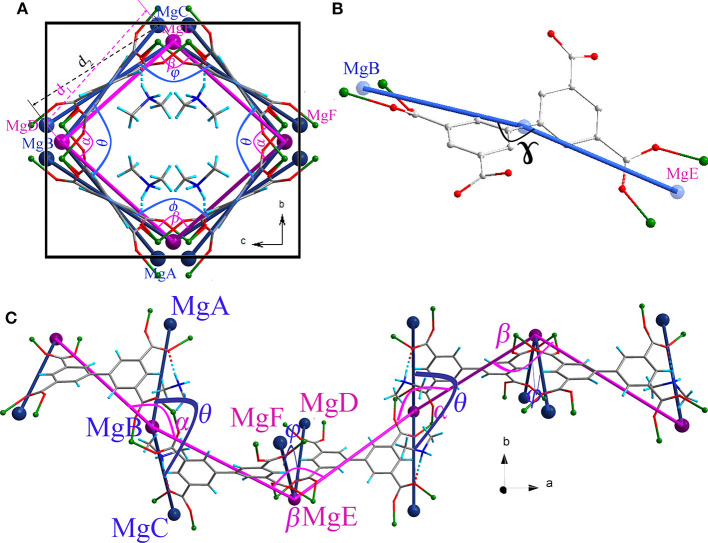
Structure of the helical chain in compound **1 (A)** viewed along the *a* axis **(C)** viewed along the *c* axis showing the hinge angle θ, ϕ, α, and β. **(B)** Structure of the ligand BPTC^4−^ showing the angle γ. (MgA, MgB, MgC, MgD, MgE, and MgF are simplified nodes located on the centers of {Mg}_2_ dinuclear units.

Thermogravimetry and variable temperature powder X-ray diffraction (PXRD) showed that compound **1** can retain framework integrity up to 363 K before irreversibly changing to another unknown phase (Figures S1, S6, S7). The helical nanotube of compound **1** is orthorhombic, but slightly deviates from a tetragonal geometry. Its crystallographic *b* axis is ~9% shorter than its *c* axis at 123 K, which may be attributed to the anisotropic interaction between the guest and the framework. Figure 2 shows a structural representation of the helical chain that can be used to analyze the flexibility of compound **1**. Angles θ, ϕ, α, β, and γ (Figure 2) are key parameters in the nanotube flexibility. Because of the orthorhombic symmetry of the space group *I*2_1_2_1_2_1_, *b* and *c* cell parameters can be expressed by simple trigonometric formulas involving the angle θ and the distance between MgB and MgC (*d*_1_), or the angle ϕ and the distance between MgD and MgE (*d*_2_), respectively (Figure 2A). Therefore, *b* = 2*d*_1_sinθ*/*2 and *c* = 2*d*_2_sinϕ*/*2. The guest [Me_2_NH_2_]^+^ cation is attached to the ligand part which is directly related to the *b* cell parameter by hydrogen bonding. In contrast, no apparent interaction can be observed for the ligand part related to the *c* cell parameter. Consequently, this host–guest interaction is highly anisotropic, straining the ligands on the framework leading to the angle θ being smaller than the angle ϕ, and *b* smaller than *c*. The anisotropic host–guest interaction may be easily modified by heating, causing the angle θ to change. Two flexible sites exist in the helical nanotube of compound **1**: (i) the torsion angle between the two phenyl groups in BPTC^4−^ ligand has been shown to be variable (Suh et al., 2006), (ii) on the turning of the helical nanotube, the Mg–Mg axis of the {Mg}_2_ dinuclear unit acts as a “knee cap,” around which the BPTC^4−^ ligands can change their angular orientations, allowing the moieties to rotate, like that observed in MIL-88 (Serre et al., 2007). These flexible features enable the angle ϕ to change oppositely with the angle θ. As a result, anisotropic thermal expansion may be anticipated in the nanotube of compound **1**.

**Figure 3:**
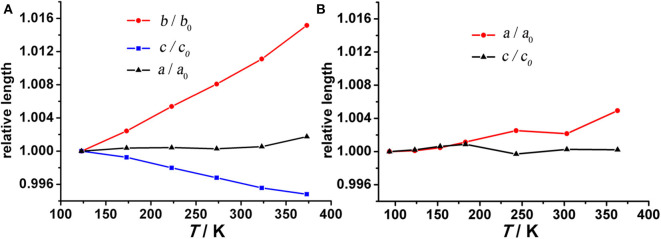
Temperature dependent unit cell parameters for **(A)** compound **1** and **(B)** compound **2**. Due to the absolute value offset, all values are normalized to 123 and 93 K for compounds **1** and **2**, respectively.

To verify this hypothesis, temperature-dependent single-crystal X-ray diffraction experiments were performed on compound **1** from 123 K to 373 K at 50 K intervals. All lattice parameters change almost linearly with increasing temperature. Interestingly, *b*- and *c*-axes change by +1.52% and −0.52% over a 250 K temperature range, while the *a*-axis remains relatively unchanged (Figure 3A). Over the measured temperature range, the thermal expansion coefficients α_*b*_ and α_*c*_ for compound **1** amount to ca. +76 × 10^−6^ K^−1^ and −26 × 10^−6^ K^−1^, respectively. The large PTE along the *b*-axis is near an order of magnitude larger than conventional PTE (Krishnan et al., 1979) and comparable to the highest values reported for solid frameworks (Goodwin et al., 2008; de Vries et al., 2011; Grobler et al., 2013). Therefore, the thermal response of compound **1** is highly anisotropic, characterized by near zero expansion along the *a*-axis and large positive expansion along the *b*-axis coupled with negative expansion along the *c*-axis, leading to an overall volumetric expansion β_*v*_ of +58 × 10^−6^ K^−1^ (Figure S8). In addition to this anisotropy, the thermal expansion behavior of compound **1** is highly desirable because its PTE/NET coefficients remain constant over wide temperature range which is beneficial for sensors and similar applications (Barrera et al., 2005; Zhou et al., 2013).

**Figure 4:**
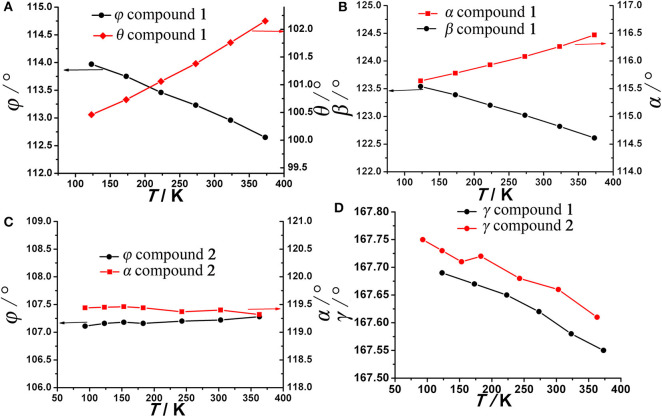
Hinge angles **(A)** φ, θ, **(B)** α, β, of compound **1 (C)** φ, α, of compound **2**, and **(D)** γ of compound **1** and **2** as a function of temperature.

**Figure 5:**
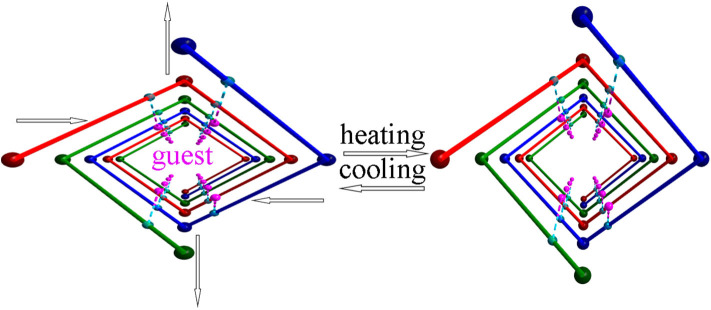
Illustration showing heat induced hinged movements of the helical nanotbe.

As anticipated, when the temperature rises from 123 to 373 K, the angle θ linearly increases from 100.47° to 102.18° whereas the angle ϕ linearly decreases from 113.95° to 112.64° (Figure 4A), which may be attributed to the hydrogen bonding interaction being significantly weakened. From 123 to 323 K, the hydrogen bond length increases from 1.91 Å to 1.98 Å (Table S1). On the other hand, as mentioned above, the helical nanotube in compound **1** consists of three parallel helices. The significant structural transformation can be illustrated in Figure 2C either. On the turning of the helix, the hinge angles α, β change according to the same trend with θ and ϕ, respectively. When the temperature increases from 123 to 373 K, the angle α linearly increases from 115.65° to 116.48° whereas the angle β linearly decreases from 123.54° to 122.61° (Figure 4B), consequently, the helical nanotube in compound **1** becomes more round on heating (Figure 5). Unexpectedly, the *a*-axis shows near zero thermal expansion rather than a conventional small positive expansion. Close examination of the helical nanotube revealed that the helical pitch corresponds to 3*a*, i.e., the projection of distance between MgB and MgE along the *a*-axis is 0.75*a* (Figure 2B). The ZTE along the *a*-axis may results from the slight rotation of phenyl rings of BPTC^4−^ ligand. The angle γ displays a slight linear decrease from 167.69° to 167.55°, when the temperature rises from 123 to 373 K (Figure 4D), counteracting the increasing of anharmonic vibrational amplitudes. To test the reversibility of the hinged movement of compound **1**, the unit cell was determined from 123 K to 373 K and then back to 123 K, where it showed an almost complete return to the original cell (Table S2).

**Figure 6:**
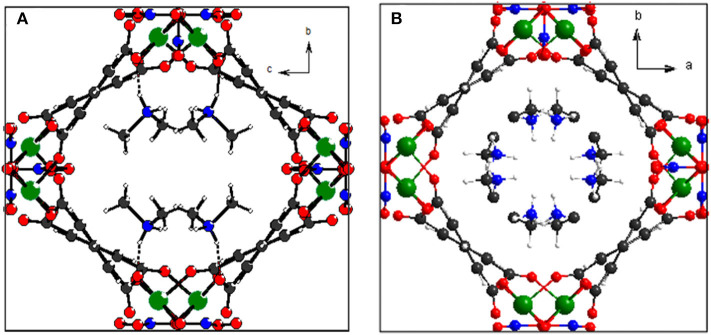
Top view of the helical nanotube in compounds **1 (A)** and **2 (B)**. Color code: Mg, green; C, gray; N, blue; O, red; H, white.

Because the anisotropic interaction between the guest and the framework play a key role in the orthorhombic phase formation and its anisotropic thermal expansion behavior, to the extreme, a tetragonal phase of the anionic framework exhibiting normal isotropic thermal expansion behavior can be expected in case of the interactions being isotropic or completely avoided. To confirm this hypothesis, we performed the similar reaction in N-Formylethylamine (NEF) and obtained the tetragonal phase of the anionic framework [EtNH_3_]·[Mg_2_(BPTC)(NO_3_)(H_2_O)] (**2**) with [EtNH_3_]^+^ cations confined in the tetragonal nanotube. Schröder et al. Hong et al., and Feng et al. have recently reported neutral frameworks with similar structure (Ibarra et al., 2011; Lin et al., 2011; Qian et al., 2012; Yang et al., 2012). In addition to μ-H_2_O, μ-NO_3_^−^ was also incorporated giving rise to the anionic framework. The IR spectra of compounds **1** and **2** are shown in Figure S9, the strong absorption at 1,573 and around 1,650 cm^−1^ for both compounds can be assigned to υ_C = O_ of BPTC^4−^. Temperature variable powder X-ray diffraction (PXRD) showed that compound **2** can retain framework integrity up to 443 K (Figure S10). In compound **2**, the [EtNH_3_]^+^ cations are confined in the center of the nanotube without notable hydrogen bond interaction with the entire framework, specifically, the nearest N···O distance between the guest and the framework is about 3.573 Å too far for the formation of hydrogen bond. As a result the nanotubes in compound **2** are considerably more round than in compound **1** (Figure 6). On the turning of the nanotube, the hinge angle θ becomes the same as ϕ with a unique value of 107.18°, and the hinge angle α also becomes identical to β with a value of 119.46° at 93 K (Figure 4C). As expected, compound **2** shows no notable structural transformation and exhibits isotropic small positive thermal expansion behavior like common materials as revealed by temperature-dependent single-crystal X-ray diffraction experiments (Figure 3B). The thermal expansion coefficients α_*a,b*_ and α_*c*_ equal ca. +18 × 10^−6^ K^−1^ and +0.8 × 10^−6^ K^−1^, respectively, in the 93–363 K temperature range. Similarly to compound **1**, the near ZTE along the *c* axis is attributed to the slight rotation of the BPTC^4−^ phenyl rings with the angle γ slightly decreasing from 167.75° to 167.61° when the temperature rises from 93 to 363 K (Figure 4D). Compared to the significant heat induced change in hinge angles observed in compound **1**, the hinge angles θ and α remain almost constant in compound **2**, implying that its framework remains almost unchanged (Figure 4C). These results strongly support that the anisotropic thermal expansion in compound **1** is because of the highly anisotropic interaction between the guest and the framework.

## Conclusion

In conclusion, an anionic framework exhibiting guest dependent phases was designed. The anisotropic thermal expansion observed in the orthorhombic phase stems from the anisotropic hydrogen bonding between the guest and the framework. This is strongly supported by the observation of normal thermal expansion of the tetragonal phase in the absence of notable hydrogen bond interaction between guest and framework. This mechanism offers a new way to transfer guest motions to host in host–guest composites. A detailed understanding of the mechanisms governing this unusual thermal expansion behavior at the molecular level may provide key information on new design principles for sensitive thermo-mechanical actuators.

## Data Availability Statement

The X-ray crystallographic coordinates for structures reported in this article have been deposited at the Cambridge Crystallographic Data Centre (CCDC), under deposition numbers CCDC 1021857-1021862 for compound 1 and 1021863-1021869 for compound 2. These data can be obtained free of charge from the Cambridge Crystallographic Data Center via www.ccdc.cam.ac.uk/data_request/cif.

## Author Contributions

ZZ: synthesized the compounds and performed PXRD measurements. Y-GH: designed this study, conducted the experiments, and wrote the manuscript. KW: designed this study. OS: designed this study and wrote the manuscript. All authors read and approved the final manuscript version to be submitted.

## Conflict of Interest

The authors declare that the research was conducted in the absence of any commercial or financial relationships that could be construed as a potential conflict of interest.
