# Supplementary material for: Anisotropic Thermal Expansion in an Anionic Framework Showing Guest-Dependent Phases
Source: Front Chem. 2020 Jun 18;8:506. doi: 10.3389/fchem.2020.00506 (PMC7314998; doi:10.3389/fchem.2020.00506)
Supplement: Supplementary file 1 [file Data_Sheet_1.docx]

Supplementary Material

# Supplementary Data

**Materials.** All reagents, except H_4_BPTC, were purchased from commercial sources and used without purification.

**Synthesis.** H_4_BPTC: H_4_BPTC was synthesized by a modified literature procedure.^[S1]^ 3,3'5,5'-tetramethylbiphenyl (1.0 g, 0.0047 mol) was oxidized using KMnO_4_ (6.5 g, 0.112 mol) in *tert*-butanol/water (v/v = 1:1; 50 mL) containing NaOH (0.4 g, 0.01 mol). Yield: 1.12 g 72.2%. Anal. Calcd (Found) for C_16_O_8_H_10_: C, 58.19 (58.10); H, 3.05 (3.09) %.

[Me_2_NH_2_]·[Mg_2_(BPTC)(NO_3_)(H_2_O)] (**1**) A mixture of Mg(NO_3_)_2_·6H_2_O (0.052 g, 0.2 mmol), H_4_BPTC (0.033 g, 0.1 mmol), 4,4'-azopyridine (0.018 g, 0.1 mmol) and HCl (1 mL 1mol/L) in DMF (5 mL) was heated at 120 °C in a sealed 20 mL glass via for one day, and cooled to room temperature. Colorless needle crystals of compound **1** were filtrated, washed using methanol and air-dried (0.041g, Yield: 80% based on Mg). Anal. Calcd (Found) for MgNC_9_O_6_H_8_: C, 43.12 (43.03); H, 3.19 (3.11); N 5.59 (5.63) %.

[EtNH_3_]·[Mg_2_(BPTC)(NO_3_)(H_2_O)] (**2**) A mixture of Mg(NO_3_)_2_·6H_2_O (0.052 g, 0.2 mmol), and H_4_BPTC (0.033 g, 0.1 mmol) in NEF (5 mL) was heated at 120 °C in a sealed 20 mL glass via for one day, and cooled to room temperature. Colorless needle crystals of compound **2** were filtrated, washed using methanol, and air-dried (0.035g, Yield: 70% based on Mg). Anal. Calcd (Found) for MgNC_9_O_6_H_8_: C, 43.12 (43.06); H, 3.19 (3.09); N 5.59 (5.51) %.

**Measurements.** Elemental analyses were performed using a Vario EL elemental analyzer. IR (KBr pellet) spectra were recorded in the range of 400–4000 cm^−1^ on a JASCO FT/IR-600 Plus spectrometer. Thermogravimetric analyses (TGA) were performed using a TG/DTA6300 system at a rate of 5 °C/min under N_2_ atmosphere. Powder X-ray diffraction (PXRD) patterns were acquired using a Rigaku 2100 diffractometer with Cu *K_α_* radiation in flat plate geometry. The temperature increased at a rate of 10 °C/min and was held constant for 5 min at each targeted temperature before measurement.

**Single-crystal X-ray diffraction.** Diffraction data were collected on a Rigaku-CCD diffractometer with Mo *K_α_* radiation. The temperature was changed at a rate of 10 °C/min and was maintained at each targeted temperature for 5 min before measurement. Structures were solved by direct method and refined by full-matrix least-squares analysis on *F*^2^ using the SHELX program. Hydrogen atoms were generated geometrically and refined in a riding model. For compound **1**, anisotropic thermal parameters were applied to all non-hydrogen atoms. Guest cations in compound **2** were isotropically refined. NO_3_^−^ and guest cations in compound **1** as well as guest cations in compound **2** were restrained by several DFIX instructions. The occupancies of *µ*-NO_3_^−^, *µ*-H_2_O and guests in all structures were refined to be consistent with the results of elemental analyses.

**Reference**

[S1]. Lin, X., Telepeni, I., Blake, A. J., Daily, A., Brown, C. M., Simmons, J. M., Zoppi, M., Walker, G. S., Thomas, K. M., Mays, T. J., Hubberstey, P., Champness, N. R., and Schröder, M. (2009) High capacity hydrogen adsorption in Cu(II) tetracarboxylate framework materials: the role of pore size, ligand functionalization, and exposed metal sites. *J. Am. Chem. Soc.* *131*, 2159-2171. doi: 10.1021/ja806624j

# Supplementary Figures and Tables

**Table S1.** Hydrogen bond lengths (Å) and angles (°) between guest and framework in compound **1** at different temperatures.

|  | T (K) | D—H | H···A | D···A | D—H···A |
| --- | --- | --- | --- | --- | --- |
| N3—H3A···O4 | 123 | 0.90 | 1.91 | 2.759(11) | 157.3 |
| N3—H3A···O4 | 173 | 0.90 | 1.93 | 2.765(11) | 154.4 |
| N3—H3A···O4 | 223 | 0.90 | 1.93 | 2.779(11) | 155.8 |
| N3—H3A···O4 | 273 | 0.90 | 1.96 | 2.799(11) | 153.8 |
| N3—H3A···O4 | 323 | 0.90 | 1.98 | 2.807(11) | 152.3 |
| N3—H3A···O4 | 373 | 0.90 | 1.95 | 2.722(14) | 143.4 |

**Table S2.** Cell parameters of compound **1** at different temperatures.

| T (K) | 123 | 173 | 223 | 273 | 323 | 373 |
| --- | --- | --- | --- | --- | --- | --- |
| *a* (Å) | 12.3226(20) | 12.3272(20) | 12.3278(18) | 12.3262(14) | 12.3293(14) | 12.3441(18) |
| *b* (Å) | 14.5559(24) | 14.5913(23) | 14.6342(19) | 14.6735(17) | 14.7175(17) | 14.7765(22) |
| *c* (Å) | 15.9349(24) | 15.9230(23) | 15.9028(19) | 15.8838(19) | 15.8642(17) | 15.8522(23) |
| *V* (Å^3^) | 2858.1878 | 2864.068 | 2868.9842 | 2872.879 | 2878.6618 | 2891.4824 |
| T (K) | cool back to 123 K |  |  |  |  |  |
| *a* (Å) | 12.323 |  |  |  |  |  |
| *b* (Å) | 14.556 |  |  |  |  |  |
| *c* (Å) | 15.935 |  |  |  |  |  |
| *V* (Å^3^) | 2858.32 |  |  |  |  |  |

**Table S3.** Cell parameters of compound **2** at different temperatures.

| T (K) | 93 | 123 | 153 | 183 | 243 | 303 | 363 |
| --- | --- | --- | --- | --- | --- | --- | --- |
| *a* (Å) | 15.2835(37) | 15.2851(37) | 15.2907(42) | 15.3009(68) | 15.3221(88) | 15.3164(64) | 15.3588(156) |
| *c* (Å) | 12.3133(30) | 12.3158(29) | 12.3210(34) | 12.3240(55) | 12.3096(66) | 12.3165(48) | 12.3160(116) |
| *V* (Å^3^) | 2876.2068 | 2877.3931 | 2880.7176 | 2880.7176 | 2889.8848 | 2889.3537 | 2905.255 |


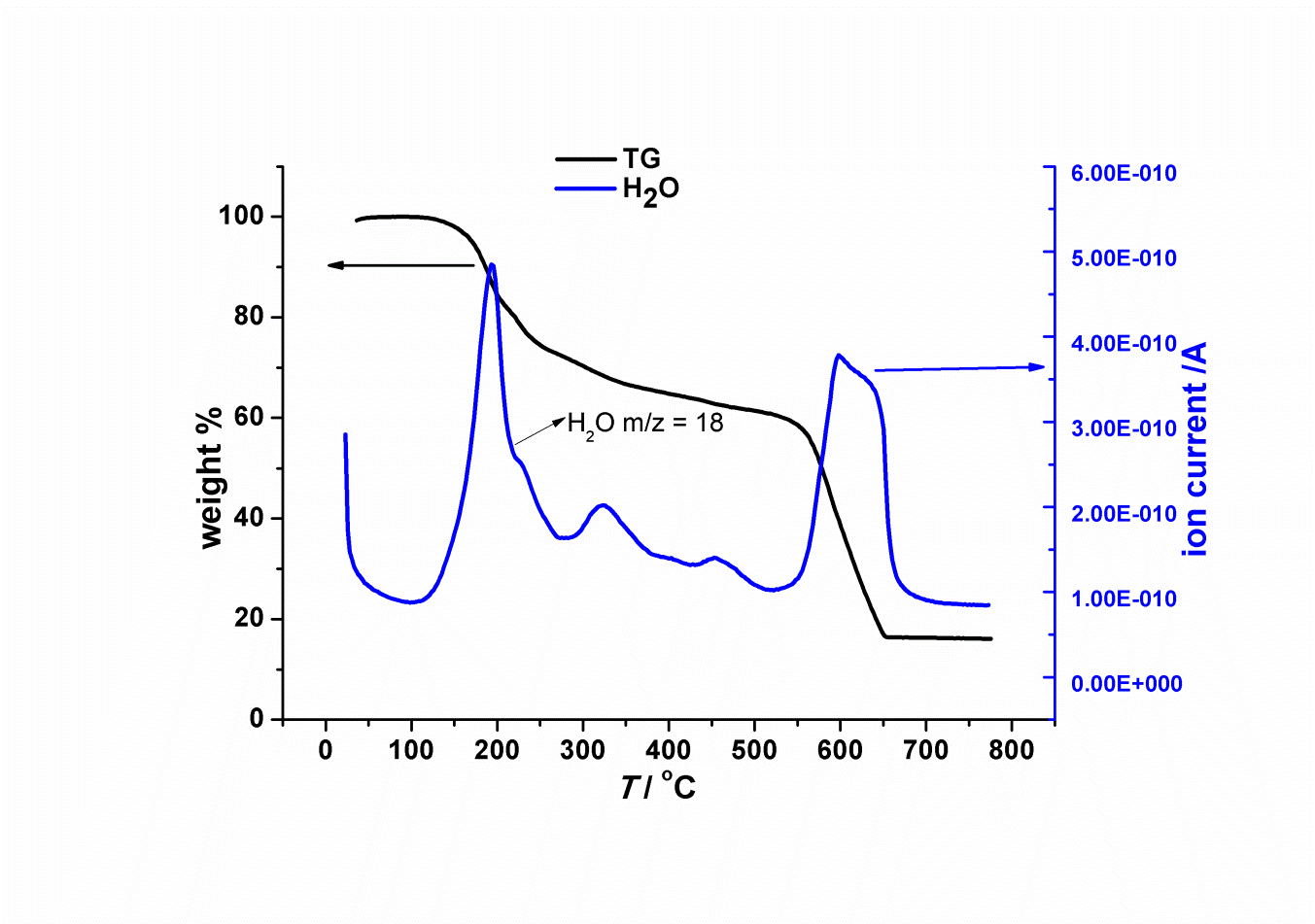


**Figure S1.** TG–MS of compound **1.** (The released H_2_O between 150 and 250 °C is *µ*-H_2_O which is statistically distributed with *µ*-NO_3_^−^ in this compound.


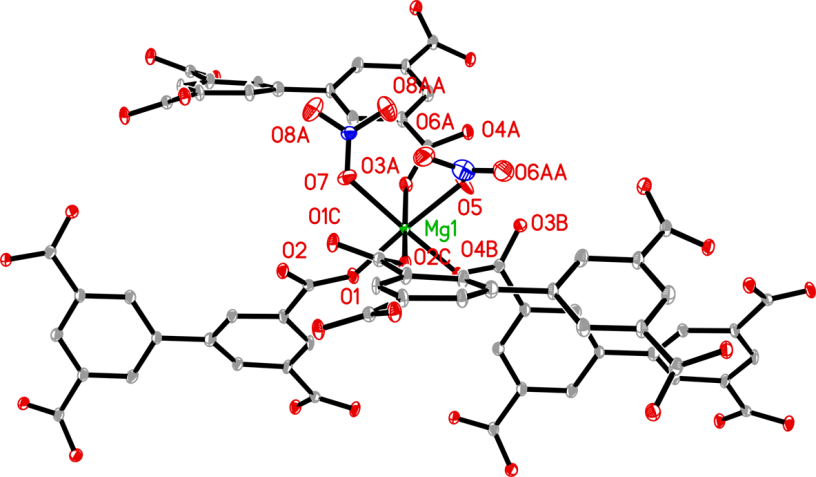


**Figure S2.** Coordination environment of Mg^2+^ ion in compound **1**. All hydrogen atoms are omitted for clarity. Symmetry codes: (A) 1−x, 1/2−y, z; (B) x−1, y, z; (C) 1/2−x, y, z; (AA) 1/2−x, y, -z. Color code: Mg: green; C: gray; N: blue; O: red.


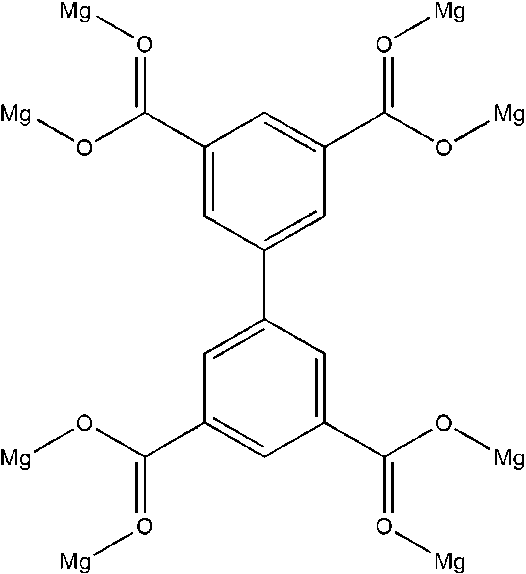


**Figure S3.** Coordination mode of BPTC^4−^ in compounds **1** and **2**.


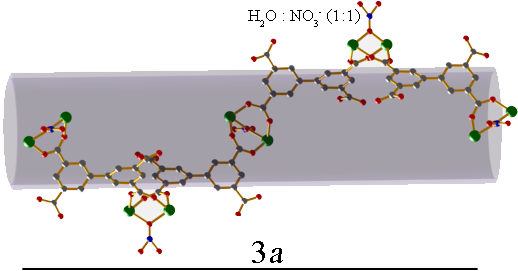


**Figure S4.** Side view of the helical chain of BPTC^4−^ linked {Mg_2_} dinuclears in compound **1** (3*a* represents the helical chain pitch). Color code: Mg: green; C: gray; N: blue; O: red.


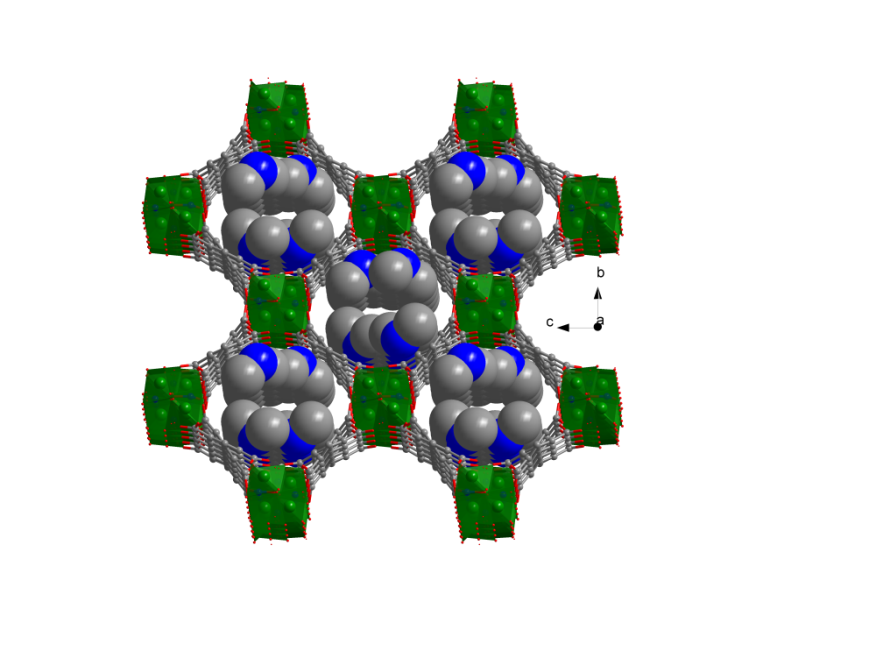


**Figure S5.** Three-dimensional anionic framework of compound **1** showing the [Me_2_NH_2_]^+^ cations in the pores. Color code: Mg green; C gray; N blue; O red.


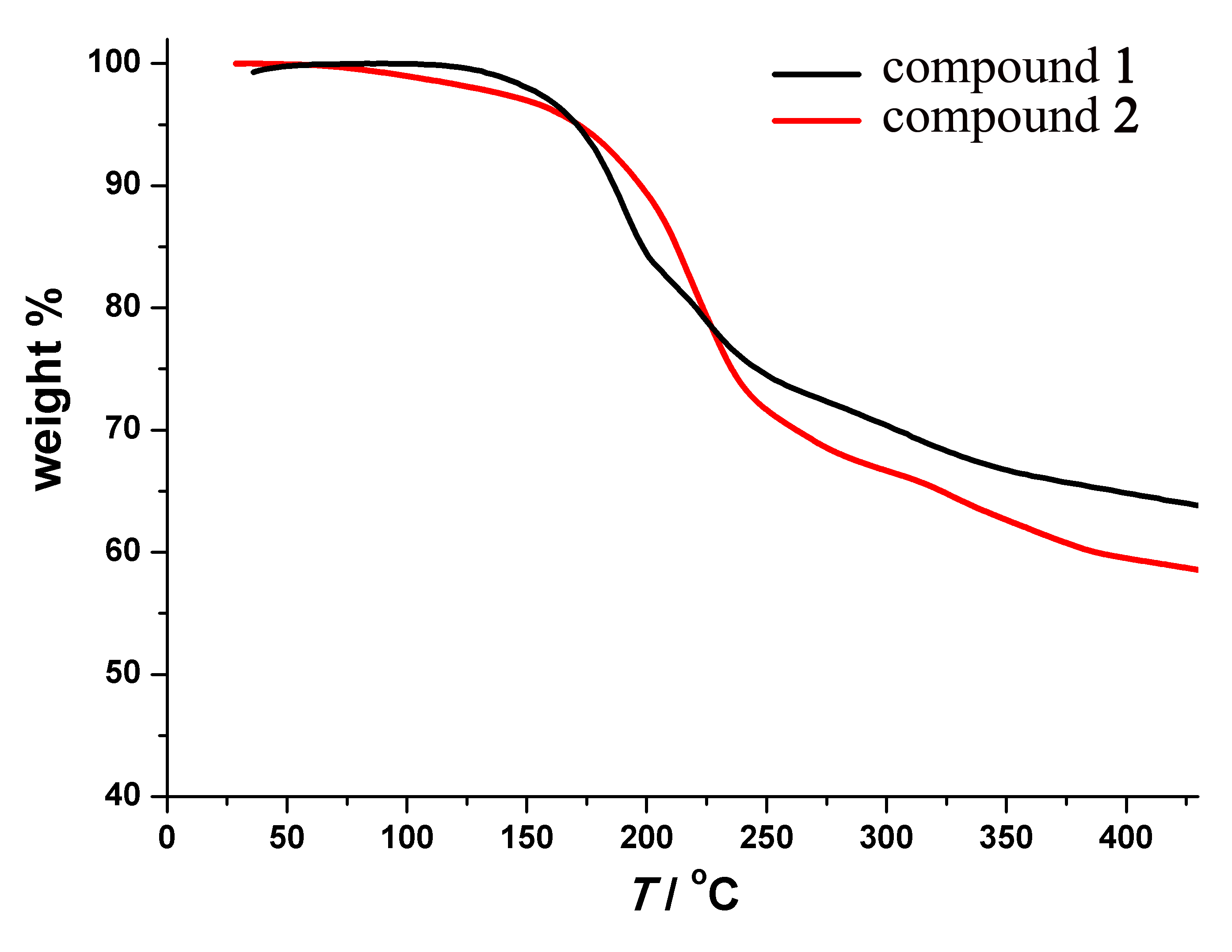


**Figure S6.** TG curves of compounds **1** and **2.**


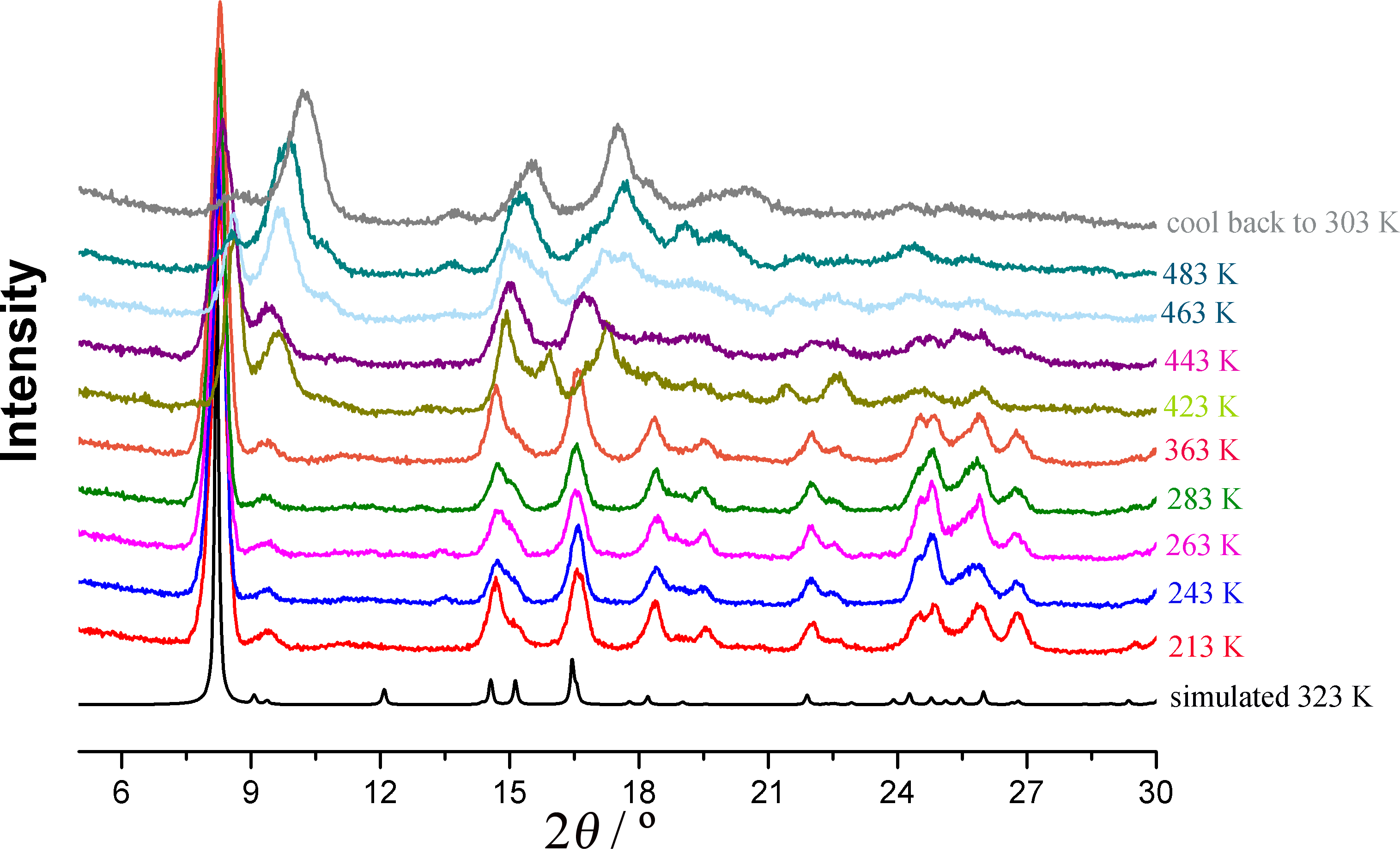


**Figure S7.** Temperature-dependent powder XRD of compound **1.**


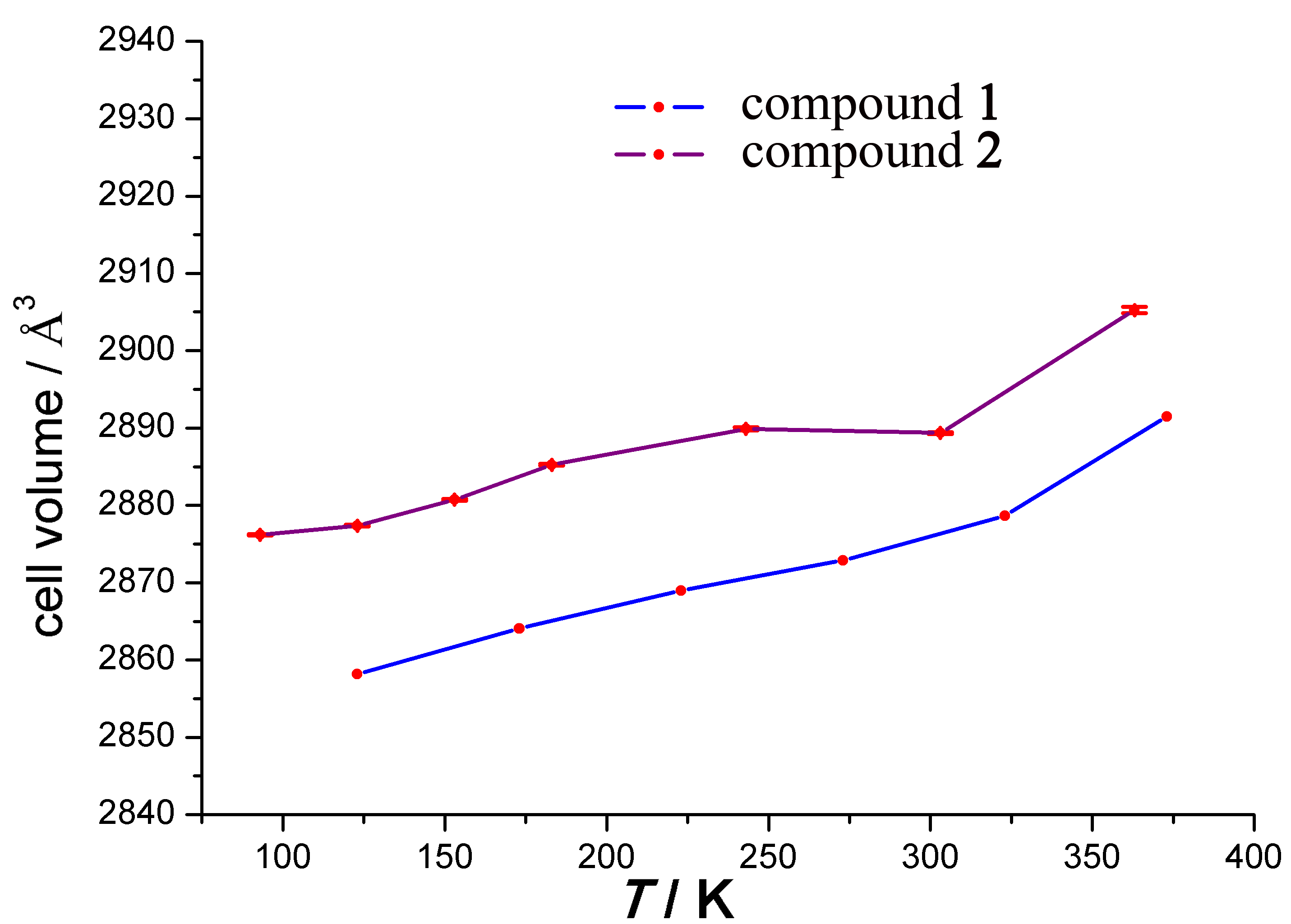


**Figure S8.** Temperature-dependent cell volume of compounds **1** and **2**.


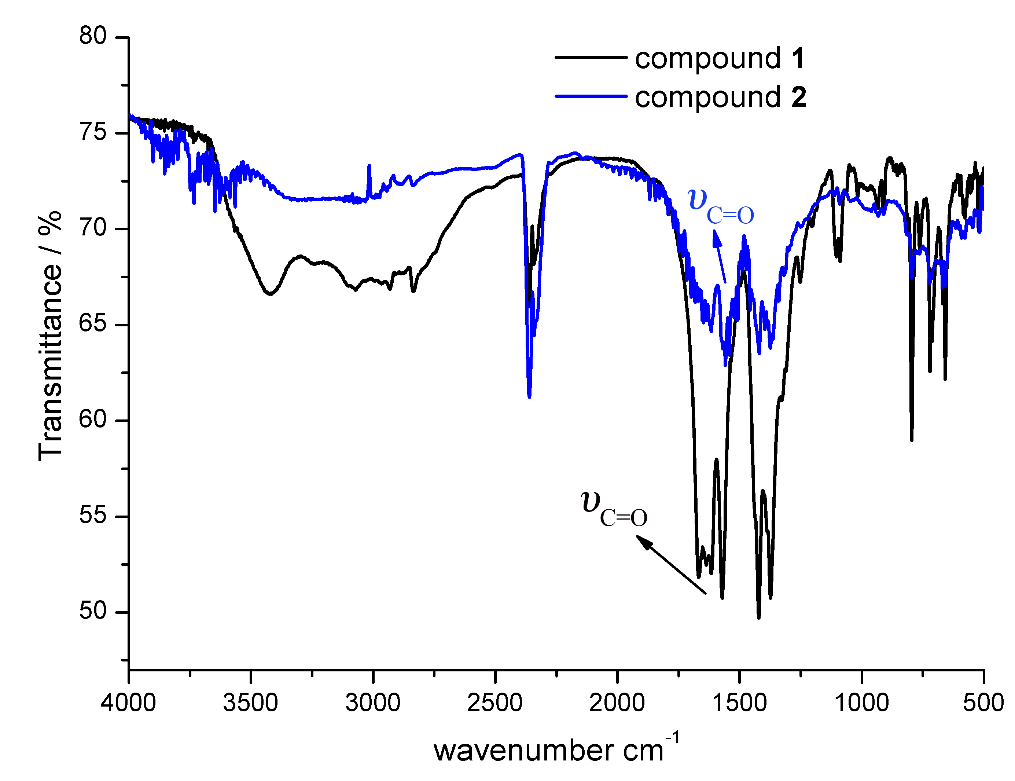


**Figure S9.** IR spectra of compounds **1**and **2.** The absorption at 1573 and around 1650 cm^−1^ for both compounds can be assigned to *ʋ*_C=O_ of BPTC^4−^.


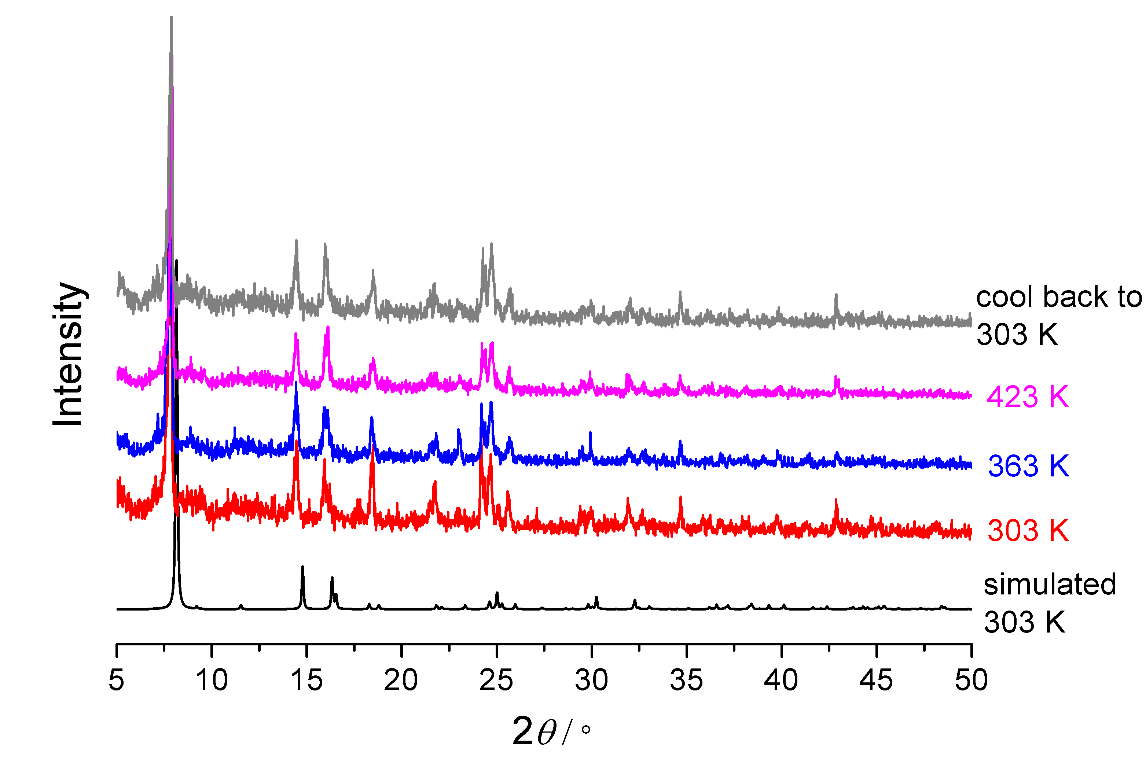


**Figure S10.** Temperature-dependent powder XRD of compound **2.**
